# Special Issue “Exercise and Neurodegenerative Disease 2.0”

**DOI:** 10.3390/jfmk10010007

**Published:** 2024-12-27

**Authors:** Grazia Maugeri, Velia D’Agata

**Affiliations:** Section of Anatomy, Histology and Movement Sciences, Department of Biomedical and Biotechnological Sciences, University of Catania, 95123 Catania, Italy; graziamaugeri@unict.it

It is well known that sedentary life is detrimental for human health; on the contrary, an active lifestyle represents an efficient instrument to guarantee and promote physical and psychological health [1,2]. According the World Health Organization (WHO), adults should perform at least 150 min of moderate-intensity activity per week, 75 min of vigorous-intensity activity, or an equivalent combination to improve their general health status [3]. There is clear evidence that physical exercise delays brain aging and counteracts neurodegenerative diseases, making the ancient principle of “*mens sana in corpore sano*” increasingly relevant. Some of these issues have been addressed and argued in the scientific contributions enclosed in the Special Issue “Exercise and Neurodegenerative Disease 2.0”; the purpose of the present Editorial is to recapitulate the contents of these articles.

The molecular mechanisms underlying the positive role of physical activity on cognitive function have been largely investigated. Some of these effects seems to be mediated by neurotrophins, such as brain-derived neurotrophic factor (BDNF), nerve growth factor (NGF), glia cell line-derived neurotrophic factor (GDNF), neurotrophin-3 (NT-3), and neurotrophin-4 (NT-4), whose expression is positively correlated to neuronal survival, neurogenesis, and synaptic plasticity [4,5,6,7,8,9,10].

Apart from the beneficial effects demonstrated by exercise for the global health in relation to physiological conditions, several studies have demonstrated the potential value of exercise in patients affected by neurodegenerative disorders [11,12,13,14], reinforcing the concept that exercise should be prescribed as an additional therapeutic intervention. In this regard, in Huntington’s disease (HD)-affected patients, physical exercise showed a positive role on gait, motor function, cognitive function, quality of life, postural stability, total body mass, fatty acid oxidative capacity, and VO2 max [15]. Moreover, another recent meta-analysis demonstrated that exercise is an efficient and safe add-on therapeutic intervention in patients not only with HD, but also with Alzheimer’s disease, multiple sclerosis, Parkinson’s disease, schizophrenia, and unipolar depression [16]. The study suggested that appropriate physical activity improved several cognitive domains with small but significant effects, without negative effects, due to exercise [16].

At the same time, physical activities can also be a major cause of injuries, such as concussion [17]. To understand the lack of neuromuscular control in the joints of healthy and injured athletes, Kakavas et al. [18] used the tensiomyography method, observing that after concussion, the muscles that control and move the knee joint are compromised. An indicator to predict autonomic nervous system (ANS) deterioration is heart rate variability (HRV), which is used to identify recovery and physical readiness among football players. Internal load was estimated via exercise cardiac load and was identified as the best solution for tracking ANS recovery in collegiate American football, when compared to external training load metrics [19]. As a clinical tool that is useful in the diagnosis of medical conditions and sport activities, three-dimensional (3D) motion analysis systems show great prospects. As such, an innovative motion capture system was used to analyze the coordination of joint motion while running in para-athletes and mild stroke patients. Furthermore, this system represents an efficient analysis tool to study normal gait [20].

This Special Issue “Exercise and Neurodegenerative Disease 2.0” comprises five articles from *Journal of Functional Morphology and Kinesiology (JFMK)* researchers. These articles provide information regarding concussion, neuromuscular control, the autonomic nervous system, Huntington’s disease, rehabilitation, motor function, brain health, synaptic plasticity, neurotrophins, neuroprotection, neurodegeneration, preventive strategy, kinematic, and lower limb joint coordination.

We hope that readers of *JFMK* enjoy reading these interesting scientific contributions.
